# Multi-Component One-Pot Synthesis and Antimicrobial Activities of 3-Methyl-1,4-diphenyl-7-thioxo-4,6,8,9-tetrahydro-pyrazolo[5,4-*b*]pyrimidino[5,4-*e*]pyridine-5-one and Related Derivatives

**DOI:** 10.3390/molecules171214464

**Published:** 2012-12-06

**Authors:** Talaat I. El-Emary, Shawkat A. Abd El-Mohsen

**Affiliations:** Department of Chemistry, Faculty of Science, Assiut University, Assiut 71516, Egypt

**Keywords:** one-pot reactions, pyrazolo[5,4-*b*]pyrimidino[5,4-*e*]pyridines, synthesis, anti-microbial activity

## Abstract

The synthesis of 3-methyl-1,4-diphenyl-7-thioxo-4,6,8,9-tetrahydropyrazolo[5,4-*b*] pyrimidino[5,4-*e*]pyridine-5-one **(6)** was achieved by two different one-pot multi-component synthesis (one-pot three-component and one-pot four component synthesis). Mono and dialkylation of **6** under different conditions gave compounds **7**–**11**. The hydrazine **12** produced from reaction of **9** with N_2_H_4_ was subjected to reactions with some aromatic aldehydes, ethyl acetoacetate, acetyl acetone, ethyl cyanoacetate and triethyl orthoformate to give **13**–**17**, respectively. Compound **12** upon reaction with CS_2_, nitrous acid, benzoin, chloroacetone and phenacyl bromide gave **18**,**20**,**21**,**22**. Alkylation of **18** with ethyl iodide, ethyl chloroacetate and phenacyl bromide gave **19a**–**c**. The antibacterial and antifungal activities of selected derivatives were evaluated.

## 1. Introduction

Multicomponent domino reactions (MDRs), particularly those performed in aqueous media, have become an increasingly useful tool for the synthesis of chemically and biologically important compounds because of their convergence, atom economy, and other suitable characteristics from the point of view of green chemistry [1,2,3,4,5,6,7,8,9,10]. Pyrazoles are excellent precursors for the synthesis of condensed polyfunctionally substituted heterocycles [11,12,13,14,15,16]. Pyrazolo-annulated heterocycles such as pyrazolopyridopyrimidines have attracted considerable interest because their derivatives display a wide range of pharmacological activities, e.g., as anticonvulsants [17], antiproliferative agents [18], anti-inflammatories and analgesic agents [19]. In addition these types of compounds are inhibitors of cyclic guanosine-3',5'-monophosphate phosphodiesterase (cGMP PDE), and are thereby agents against erectile dysfunction [20]. They also show miscellaneous biological properties such as virucidal, anticancer, fungicidal, bactericidal and vasodilatory activities [21]. For all the benefits mentioned above and as part of our program investigating syntheses using pyrazole and fused pyrazoles that have biological importance [22,23,24,25,26,27,28,29,30,31,32,33], we report herein the synthesis of pyrazolo[5,4-*b*]pyrimidino[5,4-*e*]-pyridinethiones in a one-pot four component environmental friendly method in light of recently reported methods [34,35,36,37]. 

## 2. Results and Discussion

### 2.1. Chemistry

We first describe a comparison between two methods for the construction of pyrazolo[5,4-*b*]pyrimidino[5,4-e]pyridin-5-one (**6**), a one-pot four-component domino reaction of phenyl hydrazine (**1**), 3-aminocrotononitrile (**2**), benzaldehyde (**3**) and thiobarbituric acid (**4**) in water in the presence of one equivalent of *p*-toluenesulfonic acid (*p*-TSA) (Method A) and the three-component reaction method involving 5-amino-3-methyl-1-phenylpyrazole **(5)**, benzaldehyde (**3**) and thiobarbituric acid (**4**) in presence of *p*-TSA under solvent-free conditions [35] (Method B).

**Scheme 1:**
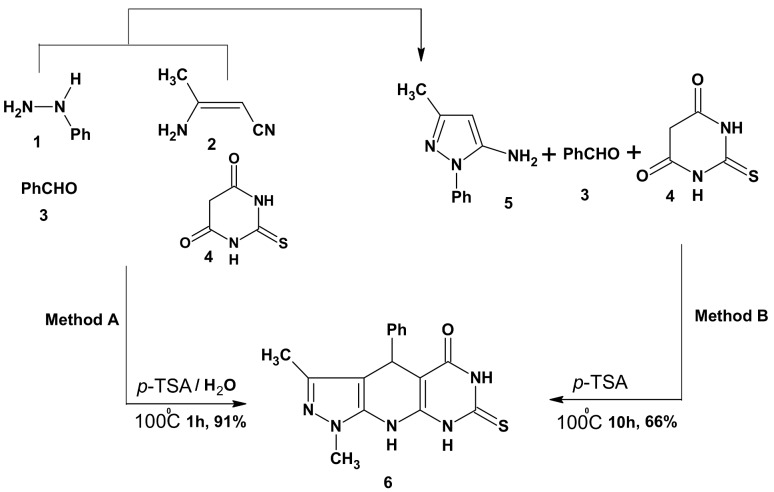
Synthetic pathways for compound **6**.

The one-pot four-component method A showed some crucial advantages, such as short reaction time, excellent yield and high purity, which makes it more efficient and broadly applicable. The percentage yield and the reaction time of the one-pot four-components method in comparison with the one-pot three-component one to produce compound **6** was found to be 91/66% and 1 h/10 h, respectively (Scheme 1).

**Scheme 2:**
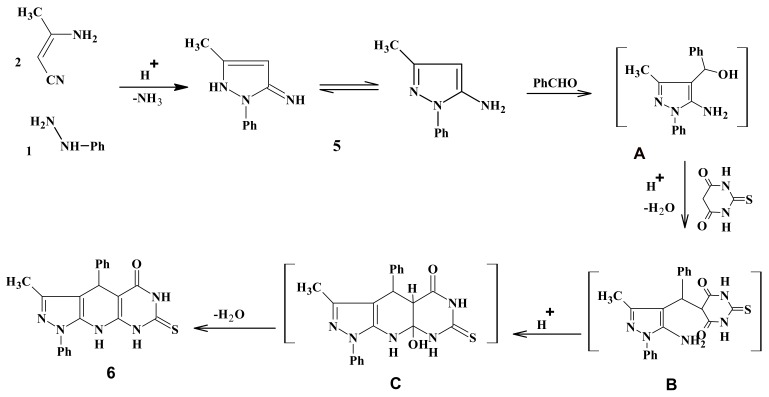
Mechanistic pathways for the formation of compound **6**.

The plausible mechanism for the formation of compound **6** is proposed (Scheme 2) and it is in agreement with that proposed in the literature [38]. The domino sequence of reactions is presumably triggered by the formation of 5-amino-3-methyl-1-phenylpyrazole (**5**) from the acid-catalyzed reaction of phenylhydrazine (**1**) with 3-aminocrotononitrile (**2**). The readily formed **5** reacts *in situ* with benzaldehyde to afford intermediate **A**. The later reacted with thiobarbituric acid under the acidic conditions to presumably furnish the intermediate **B**, which subsequently undergoes annulations leading to the final intermediate **C**. This final intermediate gave compound **6** upon losing a molecule of water.

**Scheme 3:**
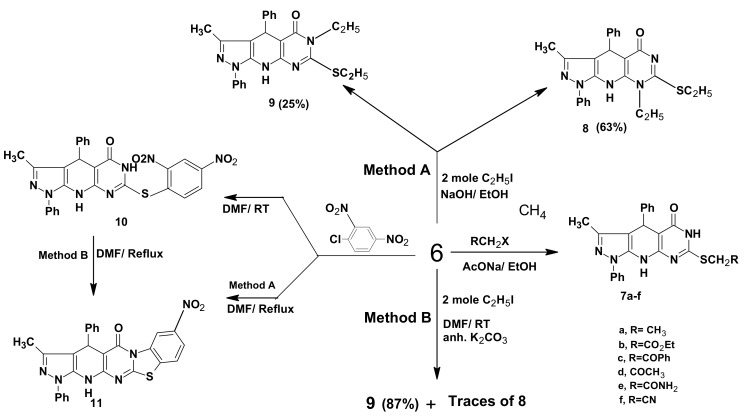
Synthetic pathways for compounds **7a**–**f**, **8**, **9**, **10** and **11**.

The structural features of compound **6** were elucidated from its spectral and analytical data. Thus, the IR spectrum revealed absorption bands at 3310, 3290, 3240, 1690, 1345 cm^−1^characteristic for three NH, C=O and C=S groups, respectively. The ^1^H-NMR (DMSO-*d_6_*) displayed three NH singlets (exchangeable with D_2_O) at 13.8, 12.2 and 9.4 ppm. A characteristic singlet peak for the pyridine CH-4 appeared at 5.57 ppm. Several alkylated derivatives were obtained from the versatile compound **6**. Thus, upon treatment of **6** with ethyl iodide, ethyl chloroacetate, phenacyl bromide, chloroacetone, chloroacetamide and chloroacetonitrile in the presence of anhydrous sodium acetate in refluxing ethanol, the S-alkylated derivatives **7a**–**f** were obtained**.** The IR spectral data of compounds **7a**–**f** displayed no absorption band for C=S and showed bands at 1280–1345 cm^−1^ confirming the S-alkylation, as well as, compounds **7b**–**e** revealed absorption bands for C=O at 1690–1735 cm^−1^. A characteristic absorption band at 2240 cm^−1^ has been observed for CN, confirming the structure of **7f**. Regioselective dialkylation reactions of compound **6** using two equivalent of ethyl iodide indicate that, in the introduction of the second ethyl group into the pyrimidine ring there is a competition between N1 or N3 and revealed that it depends on the base conditions used in the reactions. Thus, refluxing in ethanolic NaOH for 4 h (Method A) afforded the two isomeric compounds **8** and **9** in 63% and 25% yield, respectively, whereas treatment of **6** with two equivalents of ethyl iodide in DMF in the presence of anhydrous K_2_CO_3_ at room temperature (Method B) afforded in high yield the isomer **9** and only traces of the isomer **8** (Scheme 2). The reaction was monitored by TLC. The reaction mixture was chromatographically work up over silica gel using Pet. ether (b.p. 60–80 °C) and ethyl acetate (1:1) as eluent, to afforded two products in pure form. A crystalline solid, m.p. 162–164 °C obtained in the first fraction was characterized as 6-ethyl-7-ethylthio-3-methyl-1,4-diphenyl-4,6,9-trihydropyrazolo[5,4-*b*]pyrimidino[5,4-*e*] pyridine-5-one (**9**). The second fraction afforded a solid, m.p. 190–192 °C, which was identified as 8-ethyl-7-ethylthio-3-methyl-1,4-diphenyl-4,6,9-trihydropyrazolo[5,4-*b*] pyrimidino-[5,4-*e*]pyridin-5-one (**8**). By TLC comparison, data analysis and melting point determination, it was found that one of the products **9** was identical to that obtained from Method B. The chemical shift for the pyrimidinone carbonyl is markedly affected by the nature of the adjacent nitrogen [39,40,41,42,43]. The ^13^C-NMR spectral data of compound **9** showed that the δ values of the pyrimidine C=O at 165.82 ppm suggest that N-3 near to C=O is sp^3^-hybridized (pyrrole type) and different from the C=O adjacent to a sp^2^-hybridized nitrogen (pyridine type) in compound **8**, which appears at 178.14 ppm [42,43]. Moreover, reaction of **6** with highly electron withdrawing aromatic halo compounds such as 2,4-dinitrochlorobenzene in DMF at room temperature yielded the S-aryl derivative **10**, whereas, upon heating, benzothiazolo derivative **11** was obtained *via* ring closure reaction with the N3-pyrimidine ring [44]. As chemical evidence, the formation of compound **11** was achieved through heating of a sample of **10** in DMF. The structures of **10** and **11** were deduced from their satisfactory spectral and analytical data, for example, the mass spectrum of compound **11** showed its correct parent ion peak at *m/z* 506 (M^+^,100%) (Scheme 3).

**Scheme 4:**
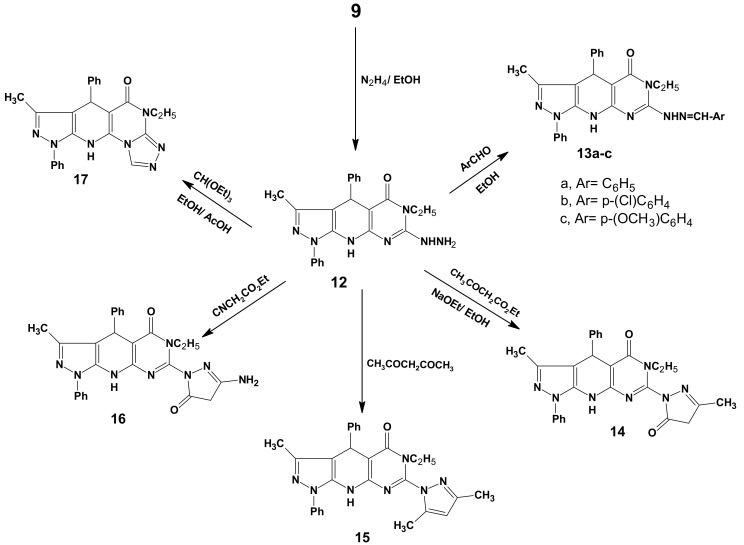
Synthetic pathways for compounds **12**–**17**.

The sulfur-free compound **12**, which was identified as 6-ethyl-7-hydrazino-3-methyl-1,4-diphenyl-4,6,9-trihydropyrazolo[5,4-*b*]pyrimidino[5,4-*e*]pyridin-5-one, was obtained in excellent yield (85%) *via* nucleophilic displacement of the thioethyl group in compound **9** with hydrazine in boiling ethanol (Scheme 4). 

The hydrazine derivative **12** was used as the key intermediate for the synthesis of some polyheterocyclic compounds. Thus, heating of compound **12** with some aromatic aldehydes namely, benzaldehyde, 4-chlorobenzaldeyde and 4-methoxybenzaldeyde in the presence of a few drops of acetic acid in ethanol resulted in the formation of the corresponding hydrazones **13a**–**c**. The structure of compounds **13a**–**c** was characterized by the disappearance of the NHNH_2_ group and revealed in each case two bands at 3425–3250 and 3170–3100 cm^−1^ assignable to two NH groups. Also, their ^1^H-NMR spectra showed the presence of the azomethine and two NH protons at 8.9–9.5 and 10.85–12.75 ppm, respectively. On the other hand, upon heating the hydrazino compound **12** with ethyl acetoacetate, acetylacetone and ethyl cyanoacetate in ethanolic sodium ethoxide solution, the *N*-pyrazolo derivatives **14**–**16** were produced. For example, the ^1^H-NMR spectrum for compound **15** revealed a singlet at 6.15 ppm due to the 4-*H*-pyrazole moiety. The ^13^C-NMR spectral data displayed two characteristic singlets at 151.12 and 152.5 ppm for C3 and C5 of the pyrazole nucleus, respectively, which was in agreement with the literature value [45]. The formation of the tetracyclic pyrazolo[5,4-*b*]1,2,4-triazolo[4',3'-2,1]pyrimidino[5,6-*e*]pyridine **(17)** was achieved upon reaction of **12** with triethyl orthoformate in ethanol in presence of a few drops of acetic acid (Scheme 4). The ^1^H-NMR spectrum of compound **17** revealed the disappearance of NHNH_2_ signals and appearance of a singlet signal at 8.55 ppm due to the triazole CH.

**Scheme 5:**
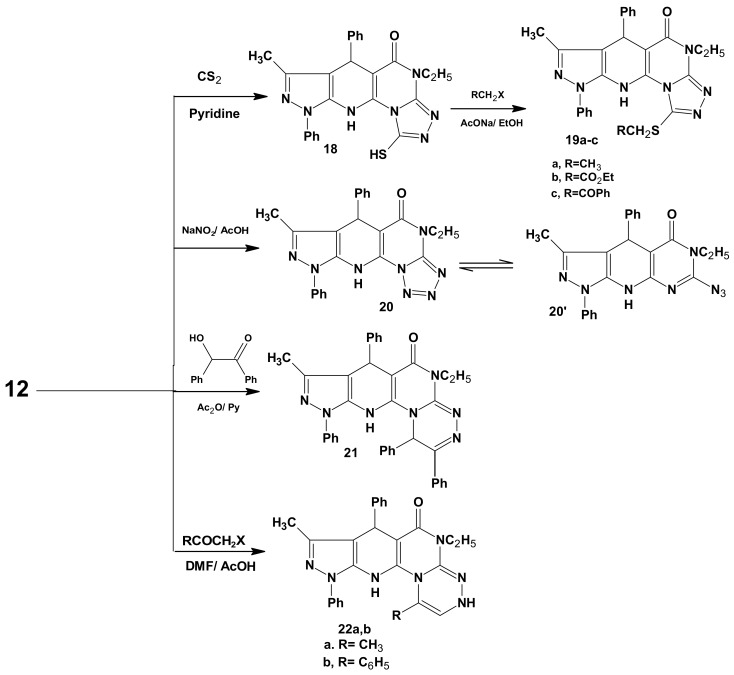
Synthetic pathways for compounds **18**–**22a**,**b**.

Furthermore, the interaction of the hydrazine **12** with CS_2_ in pyridine furnished the angular tetracyclic pyrazolo[5,4-*b*]1,2,4-triazolo[4',3'-2,1]pyrimidino[5,6-*e*]pyridin-3(2*H*)-thione (**18**) in good yield (Scheme 5). 

The thione **18** was easily converted into a series of S-alkylated derivatives **19a**–**c** upon treatment with ethyl iodide, ethyl chloroacetate and phenacyl bromide in the presence of anhydrous sodium acetate in refluxing ethanol, respectively. As well, the reaction of **11** with sodium nitrite in acetic acid at 0 °C, led to the formation of the pyrazolotetrazolopyrimidinopyridine **20** in 75% yield. This new ring system is in equilibrium with the corresponding 2-azido tautomer **20'** [46], which was confirmed by a characteristic absorption peak of the azido group at 2250 cm^−1^ in the IR. The tetracyclic pyrazolotriazinopyrimidinopyridine compounds **21** and **22a**,**b** were obtained in good to high yield when compound **12** was allowed to react with benzoin in acetic anhydride- pyridine mixture and/or with some α-haloketones (chloroacetone and phenacyl bromide) in DMF/AcOH, respectively (Scheme 5). The structures of compounds **21** and **22a**,**b** were confirmed by spectral data. For example, the ^1^H-NMR spectrum of compound **22b** revealed a singlet at 9.3 due to CH-triazine and displayed two broad singlets (D_2_O-exchangable) at 10.75 and 11.25 due to NH groups. The mass spectra of compound **22b** showed its correct parent ion peak at *m/z* 513 (M^+^, 100%).

### 2.2. Biological Results

Using the agar well-diffusion method [47], ten selected derivatives (compounds **6**, **7a**, **9**, **11**, **12**, **13b**, **17**, **19c**, **20**, and **22a**) were evaluated for their antibacterial and antifungal activities. Thus, these compounds were screened against *Staphylococcus aureus*, *Bacillus cereus*, *Micrococcus luteus* as a Gram positive bacteria and *Escherichia coil*, *Pseudomonas aeruginosa* and *Serratia marcescens* as Gram negative bacteria using chloramphenicol as control (Table 1). The MIC results indicated that three of the tested compounds (**11**, **19** and **22a**) showed significant activity against *Staphylococcus aureus* (Table 1). Compounds **12** and **19c** showed highly significant activity against *Bacillus cereus* and moderate activity against *Micrococcus luteus* (Table 1). Compound **22a** revealed moderate activity against *Staphylococcus aureus* and *Pseudomonas aeruginosa* (Table 1). The rest of tested compounds were inactive against all bacterial strains used.

The same compounds (**6**, **7a**, **9**, **11**, **12**, **13b**, **17**, **19c**, **20** and **22a**) were screened for their antifungal activities against six fungal strains: (*Candida albicans AUMC No. 418*, *Trichophyton rubrum AUMC No. 1804*, *Aspergillus flavus AUMC No. 1276*, *Fusarium oxysporum AUMC No. 5119*, *Scopulariopsis brevicaulis AUMC No. 729*, *Geotrichum candidum AUMC No. 226*) using clotrimazole as control. The results are listed in Table 2. The MIC values showed that compounds **22a**, **12** and **17** exhibit moderate to low activity against *Candida albicans AUMC No. 418.* Compounds **19c**, **12** and **11** showed moderate to low activity against *Geotrichum candidum AUMC No. 226*. Compounds **17**, **19c**, **12** and **10** revealed moderate to low activity against *Trichophyton rubrum AUMC No. 1804*. Compounds **19c**, **12** and **17** showed moderate to poor activity against *Aspergillus flavus AUMC No. 1276.* Compounds **19c**, **12** and **17** showed moderate to poor activity against *Fusarium oxysporum AUMC No. 5119.* Compounds **19c**, **12**, **17** and **11** showed moderate to poor activity against *Scopulariopsis brevicaulis AUMC No. 729*. The rest of tested compounds were inactive against all fungal strains used.

## 3. Experimental

### 3.1. General

All melting points were determined on a Kofler melting point apparatus. IR spectra were recorded on a Pye Unicam SP3-100 spectrophotometer using the KBr wafer technique. The ^1^H-NMR spectra were recorded on a Bruker ARX 200 spectrometer (200 MHz for ^1^H and 50 MHz for ^13^C) at the Faculty of Science, University of King Saoud, Saudi Arabia, Riyadh and on a Jeol LA 400 MHz (400 MHz for ^1^H, 100 MHz for the ^13^C) at Assiut University, ^1^H and ^13^C NMR chemical shifts (*δ)* were reported in parts per million (ppm) and were referenced to the solvent peak; CDCl_3_ (7.26 ppm for ^1^H and 76.90 ppm for ^13^C) and DMSO-d_6_ (2.50 ppm for ^1^H and 39.70 ppm for ^13^C). Multiplicities are represented by s (singlet), d (doublet), t (triplet), q (quartet) and m (multiplet). Coupling constants (*J*) are reported in Hertz (Hz). Mass spectra were taken on a JEOL JMS600 spectrometer at an ionizing potential of 70 eV (EI) at Assiut University. Elemental analyses were carried out using a Perkin-Elmer 240 C Micro analyzer at Assiut University and they were found to be within ± 0.4% of the theoretical values. 

### 3.2. Synthesis of 3-Methyl-1,4-diphenyl-7-thioxo-4,6,8,9-tetrahydropyrazolo[5,4-b]pyrimidino[5,4-e]pyridin-5-one *(**6**)*

Method A: A mixture of thiobarbituric acid (1.44 g, 0.01 mol), 3-methyl-1-phenyl-1*H*-pyrazol-5-amine (1.73 g, 0.01 mol), benzaldehyde (1.1 g, 0.012 mol) and *p*-TSA (1 g, 0.05 mol) was heated at 100 °C for 10 h (monitored by TLC). After cooling, the reaction mixture was washed with water (20 mL) and residue recrystallized from EtOH to afford the pure product **6** as a yellow powder. Yield: 66%. Mp 202–204 °C. IR (KBr) cm^−1^: 3240, 3290, 3310 (3NH), 1690 (C=O), 1620 (C=N), 1345 (C=S). ^1^H-NMR (DMSO-d_6_): 2.75 (s, 3H, CH_3_), 5.57 (s, 1H, CH pyridine), 7.25–7.98 (m, 10H, Ar-H), 9.45 (s, 1H, NH), 12.20 (br s, 1H, NH), 13.80 (br s, 1H, NH). ^13^C-NMR (DMSO-d_6_) δ (ppm): 11.93 (CH_3_), 33.8 (CH sp^3^), 102.11–160.12 (17C, sp^2^ carbon atoms), 161.73 (C=O), 174.45 (C=S). Anal. Calcd. For C_21_H_17_N_5_OS (387.45): C, 65.10; H, 4.42; N, 18.08; S, 8.28. Found: C, 65.15; H, 4.38; N, 18.04; S, 8.23.

Method B: A mixture of phenylhydrazine (1 mmol), 3-aminocrotononitrile (1mmol) and *p*-TSA (0.5 mmol) in water (10 mL) was added to benzaldehyde (1 mmol) and thiobarbituric acid (1 mmol) and the reaction mixture was heated under reflux at 100 °C for 1 h, after completion of the reaction (TLC), the reaction mixture cooled to room temperature, the precipitate filtered off and washed with water and recrystallized from EtOH to afford the pure product **6** as a yellow powder. Yield: 91%. All of spectral and physical data were in agreement with that described in method A. 

### 3.3. General Procedure for the Preparation of ***7a**–**f***

A mixture of **6** (3.8 g, 0.01 mol), ethyl iodide and/or α-haloketone compound (0.01 mol) in ethanol (50 mL) was refluxed in the presence of anhydrous sodium acetate (0.9 g, 0.011 mol) for 4 h. The solid product separated from the hot mixture was filtered off, washed with water and recrystallized from the proper solvent.

*7-Ethylthio-3-methyl-1,4-diphenyl-4,6,9-trihydropyrazolo[5,4-b]pyrimidino[5,4-e]pyridin-5-one* (**7a**). Yellow crystals. Yield: 72%. Mp. 135–137 °C (acetic acid). IR (KBr) cm^−1^: 3220, 3315 (2NH), 1690 (C=O), 1620 (C=N). ^1^H-NMR (DMSO-d_6_) δ (ppm): 1.55 (t, *J* = 7.4 Hz, 3H, CH_3_), 2.78 (s, 3H, CH_3_), 3.65 (q, *J* = 7.4 Hz, 2H, CH_2_), 5.55 (s, 1H, CH), 7.2–7.8 (m, 10H, Ar-H), 8.9 (s, 1H, NH), 10.2 (s, 1H, NH). ^13^C-NMR (DMSO-d_6_) δ (ppm): 12.03 (CH_3_), 15.34 (CH_3_), 24.12 (CH_2_), 33.5 (CH sp^3^), 103.15–160.12 (18C, sp^2^ carbon atoms), 161.73( C=O), Anal. Calcd. for C_23_H_21_N_5_OS (415.52): 66.48; H, 5.09; N, 16.85; S, 7.71. Found: C, 66.61; H, 5.13; N, 16.79; S, 7.68.

*Ethyl-2-(3-methyl-5-oxo-1,4-diphenyl-4,6,9-trihydro-pyrazolo[5,4-b]pyrimidino[5,4-e]pyridin-7-ylthio)acetate* (**7b**). Yellow crystals. Yield: 67%. Mp. 95–97 °C (methanol). IR (KBr) cm^−1^: 3290, 3310 (2NH), 1735 (C=O), 1690 (C=O), 1620 (C=N). ^1^H-NMR (DMSO-d_6_) δ (pp): m1.27 (t, *J* = 7.4 Hz, 3H, CH_3_), 2.80 (s, 3H, CH_3_), 3.95 (s, 2H, SCH_2_), 4.25 (q, *J* = 7.4 Hz, 2H, CH_2_), 5.52 (s, 1H, CH), 7.2–7.9 (m, 10H, Ar-H), 8.7 (s, 1H, NH), 11.1 (s, 1H, NH). ^13^C-NMR (CDCl_3_) δ (ppm): 12.03 (CH_3_), 15.34 (CH_3_), 29.12 (CH_2_), 33.55 (CH sp^3^), 61.11 (CH_2_), 103.15–160.12 (18C, sp^2^ carbon atoms), 161.73 (C=O), 170.25 (C=O ester). Anal. Calcd. for: C_25_H_23_N_5_O_3_S (473.54): C, 63.41; H, 4.90; N, 14.79; S, 6.77. Found: C, 63.49; H, 5.01; N, 14.82; S, 6.86.

*3-Methyl-7-(2-oxo-2-phenylethylthio)-1,4-diphenyl-4,6,9-trihydropyrazolo [5,4-b]pyrimidino[5,4-e]-pyridin-5-one* (**7c**). Light yellow crystals.Yield: 71%. Mp. 170–172 °C (methanol). IR (KBr) cm^−1^: 3245, 3290 (2NH), 1,685 (C=O), 1690 (C=O), 1620 (C=N); ^1^H-NMR (DMSO-d_6_) δ ppm): 2.72 (s, 3H, CH_3_), 4.19 (s, 2H, SCH_2_CO), 5.57 (s, 1H, CH), 6.9–8.6 (m, 15H, Ar-H), 8.84 (s, 1H, NH), 10.73 (s, 1H, NH); ^13^C-NMR (DMSO-d_6_) δ (ppm): 12.93 (CH_3_), 34.42 (CH sp^3^), 35.60 (CH_2_), 102.75–160.30 (18C, sp^2^ carbon atoms), 162.03 (C=O), 195.30 (C=O) Anal. Calcd. for: C_29_H_23_N_5_O_2_S (505.58): C, 68.89; H, 4.59; N, 13.85; S, 6.34. Found: C, 68.97; H, 4.64; N, 13.91; S, 6.29.

*3-Methyl-7-(2-oxopropylthio)-1,4-diphenyl-4,6,9-trihydropyrazolo[5,4-b]pyrimidino[5,4-e]pyridin-5-one* (**7d**). Yellow crystals. Yield: 65%. Mp. 223–225 °C (ethanol); IR (KBr) cm^−1^: 3195, 3290 (2NH), 1685 (C=O), 1700 (C=O); ^1^H-NMR (CDCl_3_) δ (ppm): 2.75 (s, 3H, CH_3_), 3.35 (s, 3H, COCH_3_), 4.10 (s, 2H, SCH_2_), 5.60 (s, 1H, CH), 7.35–7.95 (m, 10H, Ar-H), 9.15 (s, 1H, NH), 10.2 (s, 1H, NH). ^13^C-NMR (CDCl_3_) δ (ppm): 11.93 (CH_3_), 29.32 (CH_3_), 33.82 (CH sp^3^), 38.22 (CH_2_), 103.15–160.12 (18C, sp^2^ carbon atoms), 161.53 (C=O), 202.11 (C=O). Anal. Calcd. for: C_24_H_21_N_5_O_2_S (443.52): C, 64.99; H, 4.77; N, 15.79; S, 7.23. Found: C, 65.13; H, 4.81; N, 15.83; S, 7.19. 

*2-(3-Methyl-5-oxo-1,4-diphenyl-4,6,9-trihydropyrazolo[5,4-b]pyrimidino[5,4-e]pyridin-7-ylthio)acetamide* (**7e**). Orange crystals. Yield: 69%. Mp. 166–168 °C (DMF). IR (KBr) cm^−1^: 3420, 3400 (NH_2_), 3250, 3310 (2NH), 1685 (C=O), 1665 (C=O), 1690 (C=O). ^1^H-NMR (CDCl_3_) δ (ppm): 2.65 (s, 3H, CH_3_), 3.70 (s, 2H, SCH_2_), 5.30 (br.s, 2H, NH_2_), 6.15 (s, 1H, CH), 7.35–7.96 (m, 10H, Ar-H), 8.95 (s, 1H, NH), 10.78 (s, 1H, NH); ^13^C-NMR (CDCl_3_) δ (ppm): 12.11 (CH_3_), 30.34 (CH_2_), 34.52 (CH sp^3^), 104.85–159.90 (18C, sp^2^ carbon atoms), 162.30 (C=O), 171.45 (C=O) Anal. Calcd. for C_23_H_20_N_6_O_2_S (444.50): C, 62.15; H, 4.54; N, 18.91; S, 7.21. Found: C, 62.29; H, 4.61; N, 19.03; S, 7.15. 

*2-(3-Methyl-5-oxo-1,4-diphenyl-4,6,9-trihydropyrazolo[5,4-b]pyrimidino[5,4-e]pyridin-7-ylthio)ethanenitrile* (**7f**). Orange crystals. Yield: 61%. Mp. 147–149 °C (ethanol). IR (KBr) cm^−1^: 3215, 3200 (2NH), 2,240 (C=N), 1665 (C=O); ^1^H-NMR (DMSO-d_6_) ) δ (ppm): 2.70 (s, 3H, CH_3_), 3.90 (s, 2H, SCH_2_CO), 6.12 (s, 1H, CH), 7.55–8.22 (m, 10H, Ar-H), 9.20 (s, 1H, NH), 10.6(s, 1H, NH). ^13^C-NMR (DMSO-d_6_) δ (ppm): 12.31 (CH_3_), 15.38 (CH_2_), 33.84 (CH sp^3^), 104.80–159.95 (18C, sp^2^ carbon atoms), 161.90 (C=O). Anal. Calcd. for C_23_H_18_N_6_OS (426.49): C, 64.77; H, 4.25; N, 19.70; S, 7.52. Found: C, 64.89; H, 4.31; N, 19.61; S, 7.61. 

### 3.4. 8-Ethyl-7-ethylthio-3-methyl-1,4-diphenyl-4,6,9-trihydropyrazolo[5,4-b]pyrimidino[5,4-e]pyridin-5-one *(**8**)* and 6-Ethyl-7-ethylthio-3-methyl-1,4-diphenyl-4,6,9-trihydropyrazolo[5,4-b]pyrimidino[5,4-e]pyridin-5-one *(**9**)*

Method A: A mixture of **6** (3.8 g, 0.01 mol), ethyl iodide (3.1 g, 0.021 mol) and sodium hydroxide (0.8 g, 0.02 mol) in ethanol (50 mL) was refluxed for 4 h. Excess ethanol was distilled off and the residue obtained on cooling was found to be mixture of two products as evidenced by the TLC. The residue was subjected to column chromatography over silica gel using mixtures of pet-ether/ethyl acetate of increasing polarity of eluent to yield compound **9** as an orange solid and compound **8** as a yellow solid in the second fraction.

*8-Ethyl-7-ethylthio-3-methyl-1,4-diphenyl-4,6,9-trihydropyrazolo[5,4-b]pyrimidino[5,4-e]pyridin-5-one* (**8**). Yield: 63%. Mp. 190–192 °C. IR (KBr) cm^−1^: 3190 (NH), 1700 (C=O), ^1^H-NMR (DMSO-d_6_) δ (ppm): 1.00 (t, *J* = 7.4 Hz, 3H, NCH_2_CH_3_), 1.35 (t, *J* = 7.4 Hz, 3H, SCH_2_CH_3_), 2.68 (s, 3H, CH_3_), 3.15 (q, *J* = 7.4 Hz, 2H, NCH_2_CH_3_), 3.95 (q, *J* = 7.4 Hz, 2H, SCH_2_CH_3_), 5.65 (s, 1H, CH), 7.40–7.80 (m, 10H, Ar-H), 10.45 (s, 1H, NH). ^13^C-NMR (DMSO-d_6_) δ (ppm): 12.50 (CH_3_), 12.88 (CH_3_), 15.09 (CH_3_), 26.13 (S-CH_2_), 37.04 (CH_2_), 39.25 (CH sp^3^), 105.77–162.15 (18C, sp^2^ carbon atoms), 178.14 (C=O).

*6-Ethyl-7-ethylthio-3-methyl-1,4-diphenyl-4,6,9-trihydropyrazolo[5,4-b]pyrimidino[5,4-e]pyridin-5-one* (**9**). Yield: 25%. Mp. 162–164 °C. IR (KBr) cm^−1^: 3235 (NH), 1695 (C=O), ^1^H-NMR (DMSO-d_6_) δ (ppm): 1.32 (t, *J* = 7.4 Hz, 3H, NCH_2_CH_3_), 1.65 (t, *J* = 7.4 Hz, 3H, SCH_2_CH_3_), 2.78 (s, 3H, CH_3_), 3.45 (q, *J* = 7.4 Hz, 2H, NCH_2_CH_3_), 4.05 (q, *J* = 7.4 Hz, 2H, SCH_2_CH_3_), 5.75 (s, 1H, CH), 7.40–7.96 (m, 10H, Ar-H), 10.26 (s, 1H, NH). ^13^C-NMR (DMSO-d_6_) δ (ppm): 11.81 (CH_3_), 12.56 (CH_3_), 14.88 (CH_3_), 24.28 (S-CH_2_), 33.14 (CH_2_), 35.22 (CH sp^3^), 103.77–160.15 (18C, sp^2^ carbon atoms), 163.16 (C=O); Anal. Calcd. for: C_25_H_25_N_5_OS (443.56) C, 67.69; H, 5.68; N, 15.79; S, 7.23. Found: C, 67.81; H, 5.61; N, 15.81; S, 7.16.

Method B: A mixture of **6** (1.90 g, 0.005 mol) and ethyl iodide (1.55g, 0.01 mol) in DMF (30 mL) in the presence of anhydrous potassium carbonate (0.6 g, 0.002 mol) was stirred at room temperature for 10 h. The reaction mixture was cooled, poured onto ice cold water. The solid product separated was filtered off, washed with water and recrystallized from dioxane to yield compound **9**. All of the physical and analytical data were in agreement with those of the product obtained using method A.

### 3.5. 7-(2,4-Dinitrophenylthio)-3-methyl-1,4-diphenyl-4,6,9-trihydropyrazolo[5,4-b]pyrimidino[5,4-e] pyridin-5-one *(**10**)*

A mixture of **6** (1.14 g, 0.003 mol) and 2,4-dinitrochlorobenzene (0.6 g, 0.003 mol) in DMF (40 mL) was stirred at room temperature for 5 h. The reaction mixture was cooled to 0 °C for 2 h, a yellow fine crystals was obtained, it was filtered off dried and recrystallized from dioxane. Yield: 68%. Mp. 251–253 °C. IR (KBr) cm^−1^: 3325, 3295 (2NH), 1690 (C=O), ^1^H-NMR (DMSO-d_6_) δ (ppm): 2.75 (s, 3H, CH_3_), 5.65 (s, 1H, CH), 7.14–8.92 (m, 13H, Ar-H), 10.26 (s, 1H, NH), 11.35 (s, 1H, NH). ^13^C-NMR (DMSO-d_6_) δ (ppm): 11.90 (CH_3_), 34.35 (CH sp^3^), 103.77–161.17 (25C, sp^2^ carbon atoms), 162.99 (C=O); Anal. Calcd. for: C_27_H_19_N_7_O_5_S (553.54) C, 58.58; H, 3.46; N, 17.71; S, 5.79. Found: C, 58.96; H, 3.69; N, 17.97; S, 6.14.

### 3.6. 3-Methyl-8-nitro-1,4-diphenyl-4,6,13-trihydrobenzothiazolo[3',2'-2,1]pyrimidino[5,4-e] pyrazolo-[5,4-b]pyridin-5-one *(**11**)*

Method A: A mixture of **6** (0.38 g, 0.001 mol) and 2,4-dinitrochlorobenzene (0.2 g, 0.001 mol) in DMF (20 mL) was refluxed for 2 h. The reaction mixture was concentrated for its half volume and allowed to cool, yellowish crystals was obtained, filtered off, dried and recrystallized from acetic acid. Yield: 59%. Mp. >300 °C. IR (KBr) cm^−1^: 3180 (NH), 1685 (C=O), ^1^H-NMR (DMSO-d_6_) δ (ppm): 2.79 (s, 3H, CH_3_), 4.95 (s, 1H, CH), 7.10–7.85 (m, 13H, Ar-H), 9.95 (s, 1H, NH). ^13^C-NMR (DMSO-d_6_) δ (ppm): 12.30 (CH_3_), 34.15 (CH sp^3^), 102.57–162.11 (25C, sp^2^ carbon atoms), 163.09 (C=O); Anal. Calcd. for: C_27_H_18_N_6_O_3_S (506.53) C, 64.02; H, 3.58; N, 16.59; S, 6.33. Found: C, 64.42; H, 3.99; N, 16.91; S, 6.64. MS *m/z* (%) 506.12 (M^+^, 100).

Method B: A sample of **10** (0.5 g, 0.001 mol) was heated under reflux in DMF (30 mL) for 2 h. The separated product **11** was obtained and purified as described in method A. All physical and analytical data of the two final products obtained from both methods A and B are identical.

### 3.7. 6-Ethyl-7-hydrazino-3-methyl-1,4-diphenyl-4,6,9-trihydropyrazolo[5,4-b]pyrimidino[5,4-e]-pyridin-5-one *(**12**)*

A mixture of **9** (1.76 g, 0.004 mol) and hydrazine hydrate (15 mL) in absolute ethanol (40 mL) was refluxed for 12 h. The reaction mixture was poured onto ice. The product was isolated and crystallized from acetic acid as white needles. Yield: 85%. Mp. 280–281 °C, IR (KBr) cm^−1^: 3440, 3335, 3210 (NH, NH_2_), 1695 (C=O); ^1^H-NMR (DMSO-d_6_) δ (ppm): 1.23 (t, *J* = 7.4 Hz, 3H, NCH_2_CH_3_), 2.81 (s, 3H, CH_3_), 3.35 (q, *J* = 7.4 Hz, 2H, NCH_2_CH_3_), 4.95 (s, 2H, NH_2_, D_2_O exchangeable), 6.04 (s, 1H, CH), 7.55–8.22 (m, 10H, Ar-H), 9.82 (s, 1H, NH, D_2_O exchangeable), 10.55 (s, 1H, NH). ^13^C-NMR (DMSO-d_6_) δ (ppm): 12.21 (CH_3_), 13.01 (CH_3_), 30.58(CH_2_), 34.92 (CH sp^3^), 102.65–162.95 (18C, sp^2^ carbon atoms), 163.56 (C=O). Anal. Calcd. for: C_23_H_23_N_7_O (413.47): C, 66.81; H, 5.61; N, 23.71; Found: C, 66.95; H, 5.67; N, 23.79. 

### 3.8. General Procedure for the Preparation of ***13a**–**c***

A mixture of compound **12** (0.413 g, 0.001 mol) and the appropriate aromatic aldehyde (0.001 mol) was stirred under reflux in ethanol (30 ml) in the presence of a few drops of glacial acetic acid for 5 h. The reaction mixture was allowed to cool to room temperature, poured into water, whereby a solid formed that was filtered off and crystallized from an appropriate solvent to produce **13a**–**c** in good yields.

*6-Ethyl-3-methyl-1,4-diphenyl-4,6,9-trihydropyrazolo[5,4-b]pyrimidino[5,4-e]pyridin-5-one-7-benzaldehyde hydrazone* (**13a**). Pale white crystals from acetic acid. Yield: 70%. Mp. 268–269 °C; IR (KBr) cm^−1^: 3425, 3295 (2NH), 1675 (C=O), 1625 (C=N), ^1^H-NMR (DMSO-d_6_) δ (ppm): 1.20 (t, *J* = 7.4 Hz, 3H, NCH_2_CH_3_), 2.78 (s, 3H, CH_3_), 3.30 (q, *J* = 7.4 Hz, 2H, NCH_2_CH_3_), 6.10 (s, 1H, CH), 7.55–8.25 (m, 15H, Ar-H), 8.95 (s,1H, azomethine proton), 11.10 (brs, 1H, NH, D_2_O exchangeable), 12.75 (brs, 1H, NH, D_2_O exchangeable); ^13^C-NMR (DMSO-d_6_) δ (ppm): 11.91 (CH_3_), 12.99 (CH_3_), 30.81 (CH_2_), 35.20 (CH sp^3^), 102.65–162.95 (21C, sp^2^ carbon atoms), 162.66 (C=O), 164.11 (N=C). Anal. Calcd. for: C_30_H_27_N_7_O (501.58): C, 71.84; H, 5.43; N, 19.55; Found: C, 71.93; H, 5.59; N, 19.61.

*6-Ethyl-3-methyl-1,4-diphenyl-4,6,9-trihydropyrazolo[5,4-b]pyrimidino[5,4-e]pyridin-5-one-7-(p-chloro)benzaldehyde hydrazone* (**13b**). Pale light yellow crystals, from dioxane. Yield: 72%. Mp. 310–311 °C (dec.); IR (KBr) cm^−1^: 3320, 3170 (2NH), 1685 (C=O), 1645 (C=N), ^1^H-NMR (DMSO-d_6_) δ (ppm): 1.25 (t, *J* = 7.4 Hz, 3H, NCH_2_CH_3_), 2.88 (s, 3H, CH_3_), 3.35 (q, *J* = 7.4 Hz, 2H, NCH_2_CH_3_), 5.98 (s, 1H, CH), 7.25–8.15 (m, 14H, Ar-H), 9.15 (s,1H, azomethine proton), 10.85 (brs, 1H, NH, D_2_O exchangeable), 12.75 (brs, 1H, NH, D_2_O exchangeable); Anal. Calcd. for C_30_H_26_ClN_7_O (536.03): C, 67.22; H, 4.89; Cl, 6.61; N, 18.29; Found: C, 67.33; H, 4.94; Cl, 6.59; N, 18.41.

*6-Ethyl-3-methyl-1,4-diphenyl-4,6,9-trihydropyrazolo[5,4-b]pyrimidino[5,4-e]pyridin-5-one-7-(p-methoxy)benzaldehyde hydrazone* (**13c**). Pale white crystals from dioxane. Yield: 68%. Mp. 216–218 °C; IR (KBr)cm^−1^: 3330, 3100 (2NH), 1690 (C=O), 1635 (C=N), ^1^H-NMR (DMSO-d_6_) δ (ppm): 1.15 (t, *J* = 7.4 Hz, 3H, NCH_2_CH_3_), 2.90 (s, 3H, CH_3_), 3.30(q, *J* = 7.4 Hz, 2H, NCH_2_CH_3_),3.94 (s, 3H, OCH_3_) 5.88 (s, 1H, CH), 7.20–7.95 (m, 14H, Ar-H), 9.50 (s,1H, methylenic proton), 10.77 (brs, 1H, NH, D_2_O exchangeable), 11.45(brs, 1H, NH, D_2_O exchangeable). Anal. Calcd. for C_31_H_29_N_7_O_2_ (531.61): C, 70.04; H, 5.50; N, 18.44; Found: C, 70.14; H, 5.59; N, 18.41.

### 3.9. 6-Ethyl-3-methyl-7-(3-methyl-5-oxo(2-pyrazolinyl))-1,4-diphenyl-4,6,9-trihydropyrazolo[5,4-b]-pyrimidino[5,4-e]pyridin-5-one *(**14**)*

A solution of compound **12** (0.413 g, 0.001 mol) and ethyl acetoacetate (0.130 g, 0.001 mol) in sodium ethoxide solution [prepared by dissolving sodium metal (0.023 g, 0.001 mol) in absolute ethanol (30 mL)] was heated under reflux with stirring for 4 h. The reaction mixture was allowed to cool and poured into cold water (100 mL) and neutralized by acetic acid, whereby a solid was precipitated, which was filtered off and crystallized from chloroform. Yield: 73%. Mp. 231–233 °C; IR (KBr) cm^−1^: 3315 (NH), 1698, 1676 (2C=O), 1550 (C=N), ^1^H-NMR (CDCl_3_) δ (ppm): 1.25 (t, *J* = 7.4 Hz, 3H, NCH_2_CH_3_), 2.33 (s, 3H, CH_3_), 2.55 (s, 2H, CH_2_), 2.88 (s, 3H, CH_3_), 3.50 (q, *J* = 7.4 Hz, 2H, NCH_2_CH_3_), 5.88 (s, 1H, CH), 7.20–7.66 (m, 10H, Ar-H), 10.85(brs, 1H, NH, D_2_O exchangeable); ^13^C-NMR (CDCl_3_) δ (ppm): 11.33 (CH_3_), 12.79 (CH_3_), 24.37 (CH_3_), 30.65 (CH_2_), 34.78 (CH sp^3^), 44.65 (CH_2_ pyrazole), 103.65–162.95 (19C, sp^2^ carbon atoms), 163.66 (C=O), 164.815 (C=O pyrazole). Anal. Calcd. for: C_27_H_25_N_7_O_2_ (479.53): C, 67.63; H, 5.25; N, 20.45; Found: C, 67.71; H, 5.29; N, 20.41.

### 3.10. 7-(3,5-Dimethylpyrazolyl)-6-ethyl-3-methyl-1,4-diphenyl-4,6,9-trihydropyrazolo[5,4-b]-pyrimidine[5,4-e]pyridin-5-one *(**15**)*

A mixture of compound **12** (0.413 g, 0.001 mol) and acetylacetone (0.1 g, 0.001 mol) in absolute ethanol (30 mL) was stirred under reflux for 5 h. The reaction mixture was allowed to cool to 0 °C for 3 h. The precipitate was filtered off, dried and crystallized from ethanol as pale light crystals. Yield: 80%. Mp. 222–224 °C. IR (KBr) cm^−1^: 3225 (NH), 1700 (C=O), 1650 (C=N), ^1^H-NMR (CDCl_3_) δ (ppm): 1.27 (t, *J* = 7.4 Hz, 3H, NCH_2_CH_3_), 2.41 (s, 3H, CH_3_), 2.43 (s, 3H, CH_3_), 2.68 (s, 3H, CH_3_), 3.63 (q, *J* = 7.4 Hz, 2H, NCH_2_CH_3_), 5.77 (s, 1H, CH), 6.15 (1H, CH pyrazole), 7.20–7.82 (m, 10H, Ar-H), 10.65(s, 1H, NH); ^13^C-NMR (CDCl_3_) δ (ppm): 11.33 (CH_3_), 12.79 (CH_3_),17.62 (CH_3_), 24.37 (CH_3_), 30.65 (CH_2_), 33.66 (CH sp^3^), 102.25–160.95 (21C, sp^2^ carbon atoms), 162.90 (C=O). Anal. Calcd. for: C_28_H_27_N_7_O (477.56): C, 70.42; H, 5.70; N, 20.53; Found: C, 70.52; H, 5.79; N, 20.61.

### 3.11. 7-(3-Amino-5-oxo(2-pyrazolinyl))-6-ethyl-3-methyl-1,4-diphenyl-4,6,9-tri-hydropyrazolo[5,4-b]-pyrimidino[5,4-e]pyridin-5-one *(**16**)*

To a warmed ethanolic sodium ethoxide solution [prepared by dissolving sodium metal (0.023 g, 0.001 mol) in absolute ethanol (30 mL)] was added compound **12** (0.413 g, 0.001 mol) and ethyl cyanoacetate (0.113 g, 0.001 mol). The mixture was stirred under reflux for 12 h, the reaction mixture was allowed to cool to room temperature, then poured into cold water (100 mL) and neutralized with acetic acid. The solid product was filtered off, washed with water, ethanol, dried and crystallized from ethanol as pale brown crystals. Yield: 85%. Mp. 310–312 °C. IR (KBr) cm^−1^: 3455, 3350 (NH_2_), 3220 (NH), 1695 (C=O), 1700 (C=O), 1640 (C=N), ^1^H-NMR (CDCl_3_) δ (ppm): 1.31 (t, *J* = 7.4 Hz, 3H, NCH_2_CH_3_), 2.71 (s, 3H, CH_3_), 3.45 (s, 2H, CH_2_ pyrazole), 3.65 (q, *J* = 7.4 Hz, 2H, NCH_2_CH_3_), 6.01 (s, 1H, CH), 7.15–7.89 (m, 10H, Ar-H), 10.25 (s, 1H, NH), 12.11 (brs, NH_2_, D_2_O exchangeable). Anal. Calcd. for: C_26_H_24_N_8_O_2_ (480.52): C, 64.99; H, 5.03; N, 23.32; Found: C, 65.08; H, 5.10; N, 23.41.

### 3.12. 11-Ethyl-8-methyl-6,9-diphenyl-4,5,9,11-tetrahydropyrazolo[5,4-b]1,2,4-triazolo[4',3'-2,1]-pyrimidino[5,6-e]pyridin-10-one *(**17**)*

A mixture of compound **12** (0.413 g, 0.001 mol) and triethyl orthoformate (0.192 g, 0.0013 mol) in ethanol (30 mL) was refluxed in the presence of few drops of acetic acid for 3 h. The solid product that separated from the hot mixture was filtered off, and recrystallized from acetic acid as yellow crystals. Yield: 56%. Mp. >300 °C. IR (KBr)cm^−1^: 3315 (NH), 1695 (C=O), 1650 (C=N), ^1^H-NMR (DMSO-d_6_) δ (ppm): 1.24 (t, *J* = 7.4 Hz, 3H, NCH_2_CH_3_), 2.71 (s, 3H, CH_3_), 3.45 (q, *J* = 7.4 Hz, 2H, NCH_2_CH_3_), 6.15 (s, 1H, CH), 7.15–7.92 (m, 10H, Ar-H), 8.55 (s, 1H, CH triazole), 11.05 (s, 1H, NH). Anal. Calcd. for C_24_H_21_N_7_O (423.47): C, 68.07; H, 5.01; N, 23.15; Found: C, 68.16; H, 4.96; N, 23.22. MS *m/z* (%) 423.21 (M^+^, 100).

### 3.13. 11-Ethyl-8-methyl-6,9-diphenyl-10-oxo-4,5,9,11-tetrahydropyrazolo[5,4-b]1,2,4-triazolo[4',3'-2,1]pyrimidino[5,6-e]pyridin-3(2H)-thione *(**18**)*

A mixture of compound **12** (2.06g, 0.005 mol) and carbon disulfide (0.95 g, 0.005 mol) in ethanolic sodium hydroxide (10 mL, 10%) was heated on a water bath for 2 h. The solvent was evaporated under reduced pressure, the residue was diluted with water (30 mL), acidified with HCl. The solid product was filtered off and recrystallized from acetic acid as orange crystals. Yield: 69% Mp.285–287 °C. IR (KBr) cm^−1^: 3295 (NH), 1705 (C=O), 1620 (C=N), ^1^H-NMR (DMSO-*d_6_*). δ (ppm): 0.99 (t, *J* = 7.4 Hz, 3H, NCH_2_CH_3_), 2.45 (s, 3H, CH_3_), 3.15 (q, *J* = 7.4 Hz, 2H, NCH_2_CH_3_), 5.65 (s, 1H, CH), 7.30–7.99 (m, 10H, Ar-H), 10.75 (s, 1H, NH). Anal. Calcd. for: C_24_H_21_N_7_OS (455.53): C, 63.28; H, 4.65; N, 21.52; S, 7.04; Found: C, 63.36; H, 4.72; N, 21.62.; S, 6.98. MS *m/z* (%) 455.17 (M^+^, 100).

### 3.14. General Procedure for the Preparation of ***19a**–**c***

These compounds were synthesized following a procedure analogous to that for compounds **7a**–**f** using a mixture of **18** (0.44 g, 0.001 mol), ethyl iodide and/or α-haloketone (0.001 mol). The solid product separated from the hot mixture was filtered off, washed with water and recrystallized from ethanol.

*3-Ethylthio-11-ethyl-8-methyl-6,9-diphenyl-4,5,9,11-tetrahydropyrazolo[5,4-b]1,2,4-triazolo[4',3'-2,1]pyrimidino[5,6-e]pyridin-10-one* (**19a**). Yellow crystals. Yields: 63%. Mp. 245–247 °C. IR (KBr) cm^−1^: 3305 (NH), 1705 (C=O), 1630 (C=N), ^1^H-NMR (DMSO-d_6_) ppm δ: 1.30 (t, *J* = 7.4 Hz, 3H, NCH_2_CH_3_), 1.58 (t, *J* = 7.4 Hz, 3H, SCH_2_CH_3_), 2.69 (s, 3H, CH_3_), 3.11 (q, *J* = 7.4 Hz, 2H, NCH_2_CH_3_), 3.55( q, *J* = 7.4 Hz, 2H, SCH_2_CH_3_), 5.95 (s, 1H, CH), 7.40–7.85 (m, 10H, Ar-H), 10.30 (s, 1H, NH, D_2_O exchangeable). ^13^C-NMR (DMSO-d_6_) δ (ppm): 11.13 (CH_3_), 12.50 (CH_3_),14.65 (CH_3_), 29.75 (CH_2_), 35.60 (CH sp^3^), 42.80 (CH_2_), 103.15–160.95 (19C, sp^2^ carbon atoms), 162.20 (C=O). Anal. Calcd. for: C_26_H_25_N_7_OS (483.59): C, 64.58; H, 5.21; N, 20.27; S, 6.63; Found: C, 64.69; H, 5.30; N, 20.32; S, 6.78.

*Ethyl-3-(11-ethyl-8-methyl-6,9-dipheny-10-oxo--4,5,9,11-tetrahydro-pyrazolo[5,4-b]1,2,4-triazolo[4',3'-2,1]pyrimidino[5,6-e]pyridine)acetate* (**19b**). Yellow crystals. Yield: 79%. Mp. 189–191 °C; IR (KBr) cm^−1^: 3280 (NH), 1705 (C=O), 1735 (C=O), 1600 (C=N), ^1^H-NMR (DMSO-d_6_) δ (ppm): 1.25 (t, *J* = 7.4 Hz, 3H, NCH_2_CH_3_), 1.45 (t, *J* = 7.4 Hz, 3H, SCH_2_CH_3_), 2.70 (s, 3H, CH_3_), 3.10 (q, *J* = 7.4 Hz, 2H, NCH_2_CH_3_), 3.80 (s, 2H, SCH_2_), 4.15 (q, *J* = 7.4 Hz, 2H, SCH_2_CH_3_), 6.15 (s, 1H, CH), 7.40–7.85 (m, 10H, Ar-H), 11.03 (s, 1H, NH, D_2_O exchangeable). Anal. Calcd. for: C_28_H_27_N_7_O_3_S (541.49): C, 62.09; H, 5.02; N, 18.10; S, 5.92; Found: C, 62.23; H, 4.87; N, 18.19; S, 5.99.

*11-Ethyl-3-(2-oxo-2-phenylethylthio)--8-methyl-6,9-dipheny-10-oxo-4,5,9,11-tetrahydro-pyrazolo[5,4-b]1,2,4-triazolo[4',3'-2,1]pyrimidino[5,6-e]pyridine-10-one* (**19c**). Pale yellow crystals. Yield: 68%. Mp. 272–274 °C. IR (KBr) cm^−1^: 3290 (NH), 1705 (C=O), 1690 (br, C=O), 1600 (C=N), ^1^H-NMR (DMSO-d_6_) δ (ppm): 1.18 (t, *J* = 7.4 Hz, 3H, NCH_2_CH_3_), 2.77 (s, 3H, CH_3_), 3.32 (q, *J* = 7.4 Hz, 2H, NCH_2_CH_3_), 4.35 (s, 2H, SCH_2_), 5.95 (s, 1H, CH), 7.20–8.25 (m, 15H, Ar-H), 10.80 (s, 1H, NH, D_2_O exchangeable). Anal. Calcd. for C_32_H_27_N_7_O_2_S (573.67): C, 67.01; H, 4.74; N, 17.09; S, 5.59; Found: C, 67.21; H, 4.83; N, 17.23.; S, 5.49. MS *m/z* (%) 573.57 (M^+^, 100).

### 3.15. 4-Ethyl-7-methyl-6,9-diphenyl-4,6,10,11-tetrahydropyrazolo[5,4-b]1,2,3,4-tetraazolo[1',5'-1,2]-pyrimidino[5,6-e]pyridin-5-one *(**20**)*

A solution of sodium nitrite (0.07 g, 0.001 mol) in the least amount of water was added dropwise to an ice-cold solution of compound **12** (0.413 g, 0.001 mol) in acetic acid (10 mL) kept in an ice bath at −5 °C. The reaction mixture was allowed to stand overnight at room temperature, then it was poured into water (100 mL). The precipitate that formed was filtered off and crystallized from dioxane. It separated as pale yellow needles. Yield: 75%. Mp. > 300 °C; IR (KBr) cm^−1^: 3350 (NH), 2250 (N_3_), 1695 (C=O), 1620 (C=N). ^1^H-NMR (DMSO-d_6_) δ (ppm): 1.33 (t, *J* = 7.4 Hz, 3H, NCH_2_CH_3_), 2.60 (s, 3H, CH_3_), 3.54 (q, *J* = 7.4 Hz, 2H, NCH_2_CH_3_), 5.85 (s, 1H, CH), 7.20–7.90 (m, 10H, Ar-H), 11.25 (s, 1H, NH, D_2_O exchangeable). ^13^C-NMR (DMSO-d_6_) δ (ppm ): 11.03 (CH_3_), 12.35 (CH_3_), 33.45 (CH sp^3^), 40.65 (CH_2_), 101.75–160.15 (18C, sp^2^ carbon atoms), 163.30 (C=O). Anal. Calcd. for C_23_H_20_N_8_O (424.46): C, 65.08; H, 4.75; N, 26.40; Found: C, 65.19; H, 4.83; N, 26.55.

### 3.16. 5-Ethyl-8-methyl-1,2,7,10-tetraphenyl-5,7,11,12-tetrahydro-1H-pyrazolo[5,4-b]1,2,4-triazino[4',3'-2,1]-pyrimidino[5,6-e]pyridin-6-one *(**21**)*

A mixture of compound **12** (0.413g, 0.001 mol) and benzoin (0.21 g, 0.001 mol) was heated under reflux in a mixture of pyridine and acetic anhydride (20 mL) (1:1) for 5 h. The reaction mixture was allowed to cool, poured onto ice cold water and neutralized with dilute HCl, The solid product was filtered off and recrystallized from acetic as pale gray needles. Yield: 55%. Mp. > 300 °C; IR (KBr) cm^−1^: 3270 (NH), 1690 (C=O), 1640(C=N). ^1^H-NMR (DMSO-d_6_) δ (ppm): 1.35 (t, *J* = 7.4 Hz, 3H, NCH_2_CH_3_), 2.40 (s, 3H, CH_3_), 3.15 (s, 1H, CH), 3.35 (q, *J* = 7.4 Hz, 2H, NCH_2_CH_3_), 6.05 (s, 1H, CH), 7.20–7.90 (m, 20H, Ar-H), 10.40 (s, 1H, NH, D_2_O exchangeable). ^13^C-NMR (DMSO-d_6_) δ (ppm): 11.03 (CH_3_), 12.55 (CH_3_), 32.35 (CH sp^3^), 38.70 (CH_2_), 44.55 (CH sp^3^), 105.80–161.85 (31C, sp^2^ carbon atoms), 162.90 (C=O). Anal. Calcd. for C_37_H_31_N_7_O (589.66): C, 75.35; H, 5.82; N, 16.19; Found: C, 75.73; H, 6.12; N, 16.53.

### 3.17. General Procedure for the Preparation of ***22a**,**b***

A mixture of compound **12** (0.001 mol) with chloroacetone or phenacyl bromide (0.001 mol) in DMF (30 mL) and drops of glacial acid (0.2 mL) was heated in 90 °C for 10 h. The solid that precipitated upon cooling was filtered off and crystallized from ethanol.

*5-Ethyl-1,8-dimethyl-7,10-diphenyl-5,7,11,12-tetrahydro-3H-pyrazolo[5,4-b]1,2,4-triazino[4',3'-2,1]-pyrimidino[5,6-e]pyridin-6-one* (**22a**). Pale white crystals. Yield: 51%. Mp. 241–243 °C (dec.). IR (KBr) cm^−1^: 3310, 3290 (2NH), 1685 (C=O) 1630 (C=N), ^1^H-NMR (DMSO-d_6_) δ (ppm): 1.20 (t, *J* = 7.4 Hz, 3H, NCH_2_CH_3_), 2.33 (s, 3H, CH_3_), 2.65 (s, 3H, CH_3_), 3.54 (q, *J* = 7.4 Hz, 2H, NCH_2_CH_3_), 5.70 (s, 1H, CH), 7.15–7.95 (m, 10H, Ar-H), 9.25 (s, 1H, triazine), 10.80 (s, 1H, NH, D_2_O exchangeable), 10.95 (s, 1H, NH, D_2_O exchangeable). ^13^C-NMR (DMSO-d_6_) δ (ppm ): 11.90 (CH_3_), 12.45 (CH_3_), 16.55 (CH^3^), 30.98 (CH_2_), 34.95 (CH sp^3^), 36.15 (CH sp^3^), 104.90–161.85 (19C, sp^2^ carbon atoms), 162.90 (C=O). Anal. Calcd. for C_26_H_25_N_7_O (451.52): C, 69.16; H, 5.58; N, 21.71; Found: C, 69.24; H, 5.73; N, 21.81.

*5-Ethyl-8-methyl-1,7,10-triphenyl-5,7,11,12-tetrahydro-3H-pyrazolo[5,4-b]1,2,4-triazino[4',3'-2,1]-pyrimidino[5,6-e]pyridin-6-one* (**22b**). Pale white crystals. Yield: 63%. Mp. >300 °C, IR (KBr) cm^−1^: 3400, 3390 (2NH), 1685 (C=O) 1630 (C=N), ^1^H-NMR (DMSO-d_6_) δ (ppm): 1.19 (t, *J* = 7.4 Hz, 3H, NCH_2_CH_3_), 2.55 (s, 3H, CH_3_), 4.04 (q, *J* = 7.4 Hz, 2H, NCH_2_CH_3_), 6.10 (s, 1H, CH), 7.15–8.15 (m, 15H, Ar-H), 9.30 (s, 1H, triazine), 10.75 (s, 1H, NH, D_2_O exchangeable), 11.25 (s, 1H, NH, D_2_O exchangeable). Anal. Calcd. for: C_31_H_27_N_7_O (513.59): C, 72.50; H, 5.30; N, 19.09; Found: C, 72.64; H, 5.41; N, 19.16. MS *m/z* (%) 513.48 (M^+^, 100).

### 3.18. Antimicrobial Activity

The antimicrobial activity of 10 new chemical compounds was tested in *vitro* against six bacterial species obtained from contaminated soil, water and food substances (*Staphylococcus aureus* [AUMC No. B-54], *Bacillus cereus* [AUMC No. B-52], *Micrococcus luteus (+ve)* [AUMC NoB-112], *Escherichia coli* [AUMC No. B-53], *Pseudomonas aeruginosa* [AUMC No. B-73] and *Serratia marcescens* [AUMC No. B-55]. They were also tested against six fungal species which are involved in human and animal diseases (*Trichophyton rubrum* [AUMC No. 1804], *Candida albicans* [AUMC No. 418], *Geotrichum candidum* [AUMC No. 226], *Scopulariopsis brevicaulis*[AUMC No. 729] and *Aspergillus flavus*[AUMC No. 3214] or plant diseases (*Fusarium oxysporum* [AUMC No. 5119]. These strains are common contaminants of the environment in Egypt and some of all microbial strains were kindly provided by the Assiut University Mycological Centre (AUMC). To prepare inocula for bioassay, bacterial strains were individually cultured for 48 h in 100 mL conical flasks containing 30 mL nutrient broth medium. Fungi were grown for 7 days in 100 mL conicals containing 30 mL Sabouraud's dextrose broth. Bioassay was done in 10 cm sterile plastic Petri plates in which microbial suspension (1 mL/plate) and 15 mL appropriate agar medium (15 mL/plate) were poured. Nutrient agar and Sabouraud's dextrose agar were respectively used for bacteria and fungi. After solidification of the media, 5 mm diameter cavities were cut in the solidified agar (4 cavities/plate) using sterile cork borer. Chemical compounds dissolved in DMSO at 2%w/v (=20 mg/mL) were pipetted in the cavities (20 µL/cavity). Cultures were then incubated at 28 °C for 48 h in case of bacteria and up to 7 days in case of fungi. Results were read as the diameter (in mm) of inhibition zone around cavities [29]. To determine the minimum inhibitory concentrations (MICs), chemical compounds giving positive results were diluted with DMSO to prepare a series of descending concentrations down to 0.02 mg/mL. Diluted chemicals were similarly assayed as mentioned before and the least concentration (below which no activity) was recorded as the MIC.

## 4. Conclusions

In conclusion, we have reported the synthesis of some novel heterocyclic pyrazolo-pyrimidinopyridines and related compounds. Ten of the newly synthesized compounds have been screened for their biological activities against three Gram positive, three Gram negative bacteria, as well as six fungal strains. Most of the tested compounds showed activities against the strains used. Compound **19c** proved to be the most potent compound of all those used.

## Figures and Tables

**Table 1:** Antibacerial activity data [inhibition zone in mm/MICs (in mM).

Compound No.	Diameter of the inihibition zone (mm) MIC (mM)					
	*S. aureus* *AUMC* *B.54*	*S. cereus* *AUMC* *B.52*	*M. luteus AUMC* *B.112*	*E. coli* *AUMC* *B.53*	*p. aeruginosa AUMC* *B.73*	*S. marcescens AUMC* *B.55*
**6**	-	-	-	-	-	-
**7a**	-	-	-	-	-	-
**9**	-	-	-	-	-	-
**11**	**8 (2.5)**	-	-	-	-	-
**12**	-	**10 (1.25)**	**18 (20)**	-	-	-
**13b**	-	-	-	-	-	-
**17**	-	-	-	-	-	-
**19c**	**8 (1.25 )**	**10 (0.15)**	**10 (5)**	-	-	-
**20**	-	-	-	-	-	-
**22a**	**8 (5)**	-	-	-	**11 (20)**	-
**CHL**	**10 (0.08)**	**12 (1.25)**	**12 (2.5)**	**10 (0.08)**	**12 (0.3)**	**13 (1.25)**

CHL = chloramphenicol as control.

**Table 2:** Antifungal activity data [inhibition zone in mm/ MICs (in mM).

Compound No.	Diameter of the inihibition zone (mm) MIC (mM)					
	*C. albicans* *AUMC* *418*	*G.candidum* *AUMC* *226*	*T. rubrum* *AUMC* *1804*	*A.flavus* *AUMC* *3214*	*F. oxysporum* *AUMC* *5119*	*S. brevicaylis* *AUMC* *729*
**6**	-	-	-	-	-	-
**7a**	-	-	-	-	-	-
**9**	-	-	-	-	-	-
**11**	-	**11 (20)**	**8 (20)**	-	-	**10 (20)**
**12**	**9 (2.5)**	**15 (5)**	**10 (10)**	**10 (2.5)**	**16 (20)**	**20 (5)**
**13b**	-	-	-	-	-	-
**17**	**10 (20)**	-	**24p.i (20)**	**10 (20)**	**8 (20)**	**12 (10)**
**19c**	**9 (2.5)**	**16 (2.5)**	**12 (5)**	**14 (2.5)**	**10 (10)**	**28 (5)**
**20**	-	-	-	-	-	-
**22a**	**10 (0.6)**	-	-	-	-	-
**CLO**	**12 (0.08)**	**14 (0.08)**	**35 (0.08)**	**15 (0.15)**	**14 (0.15)**	**24 p.i (0.3)**

CLO = Clotrimazole as control; p.i = Partial inhibition.

## Supplementary Materials

Supplementary materials can be accessed at: http://www.mdpi.com/1420-3049/17/12/14464/s1.
