# Supplementary material for: Multi-Component One-Pot Synthesis and Antimicrobial Activities of 3-Methyl-1,4-diphenyl-7-thioxo-4,6,8,9-tetrahydro-pyrazolo[5,4-b]pyrimidino[5,4-e]pyridine-5-one and Related Derivatives
Source: Molecules. 2012 Dec 6;17(12):14464–83. doi: 10.3390/molecules171214464 (PMC6268947; doi:10.3390/molecules171214464)

^{1}H-NMR spectrum of compound 6

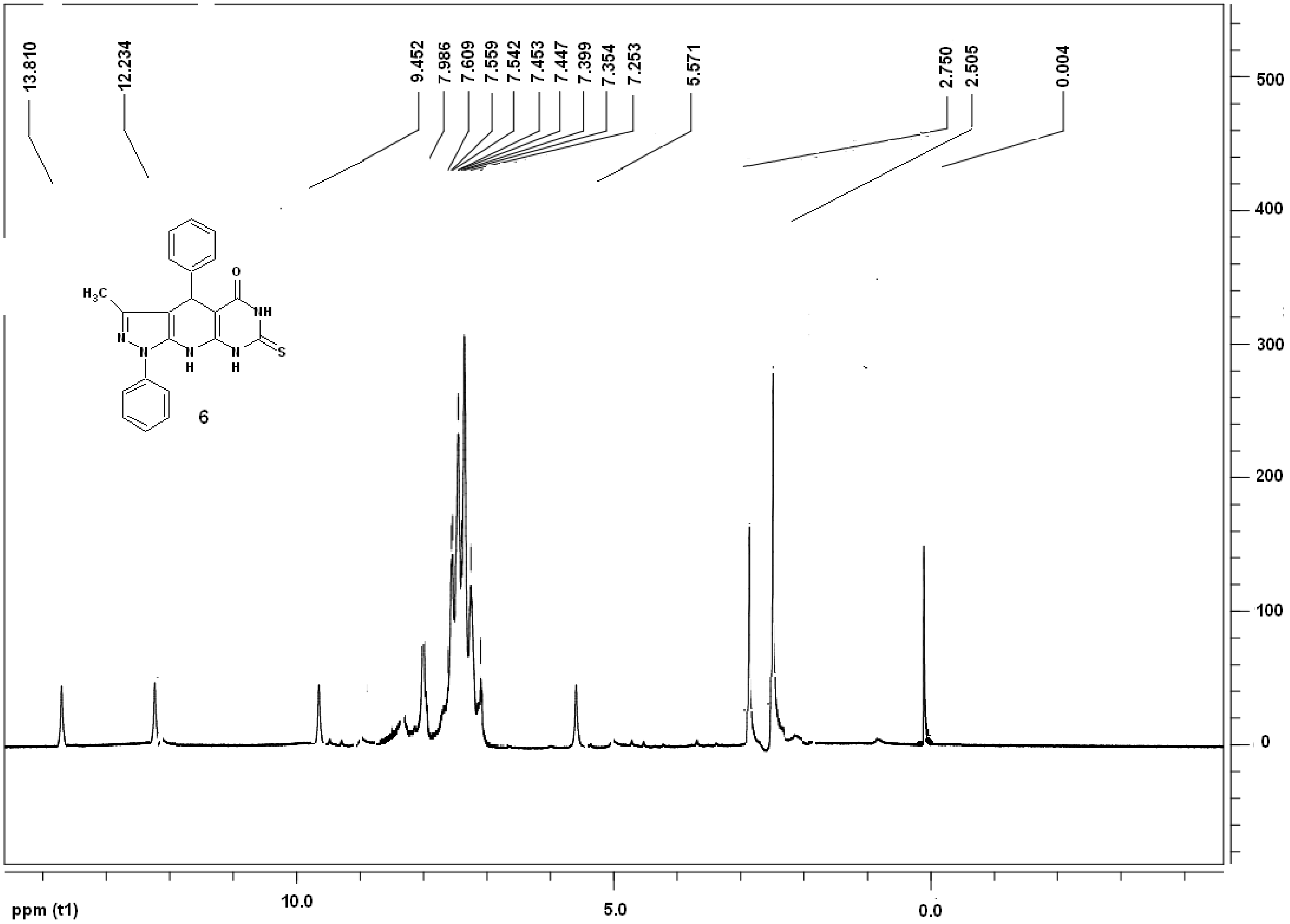

^{1}H-NMR spectrum of compound 7d

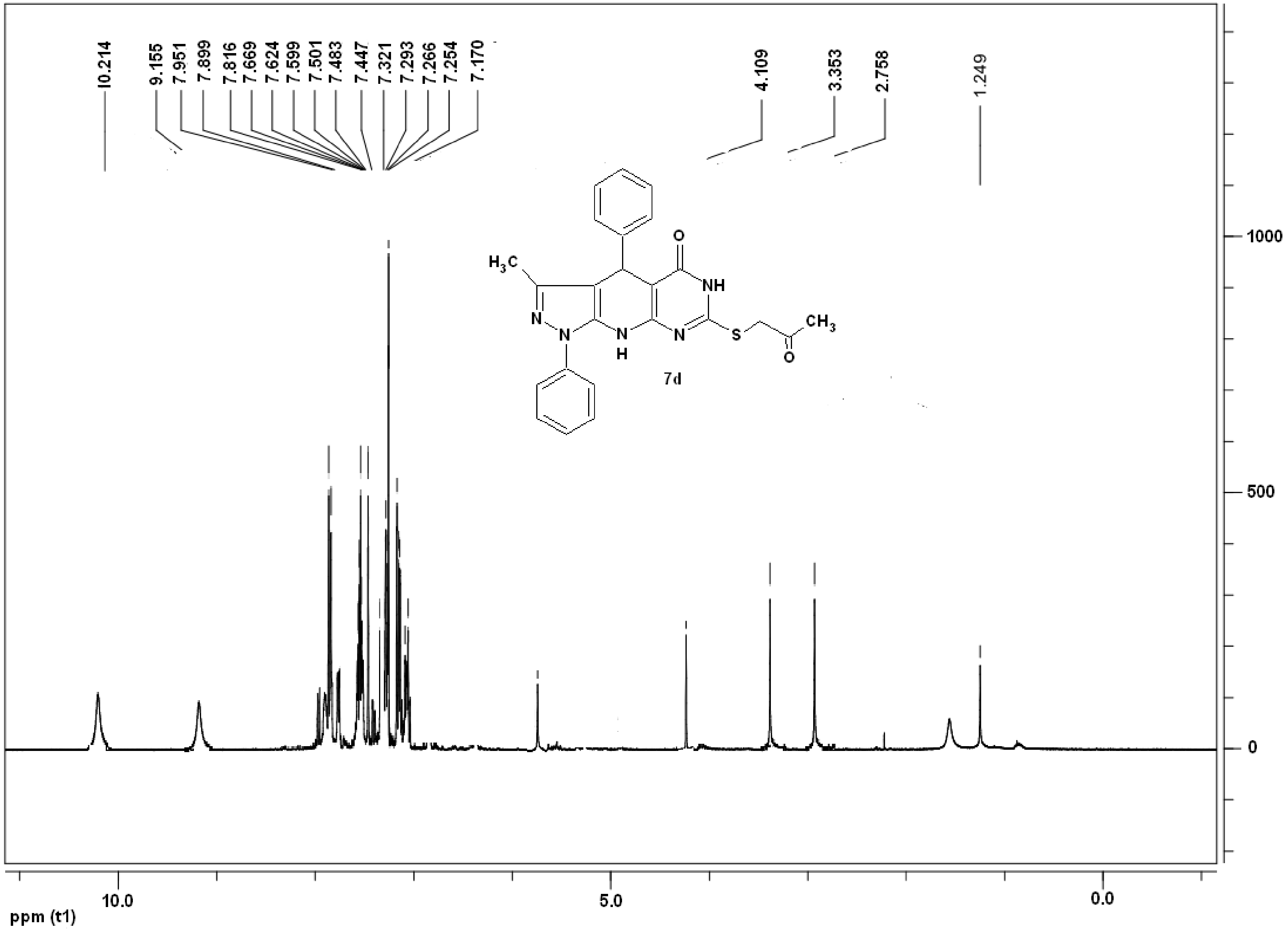

IR spectrum of compound 7f

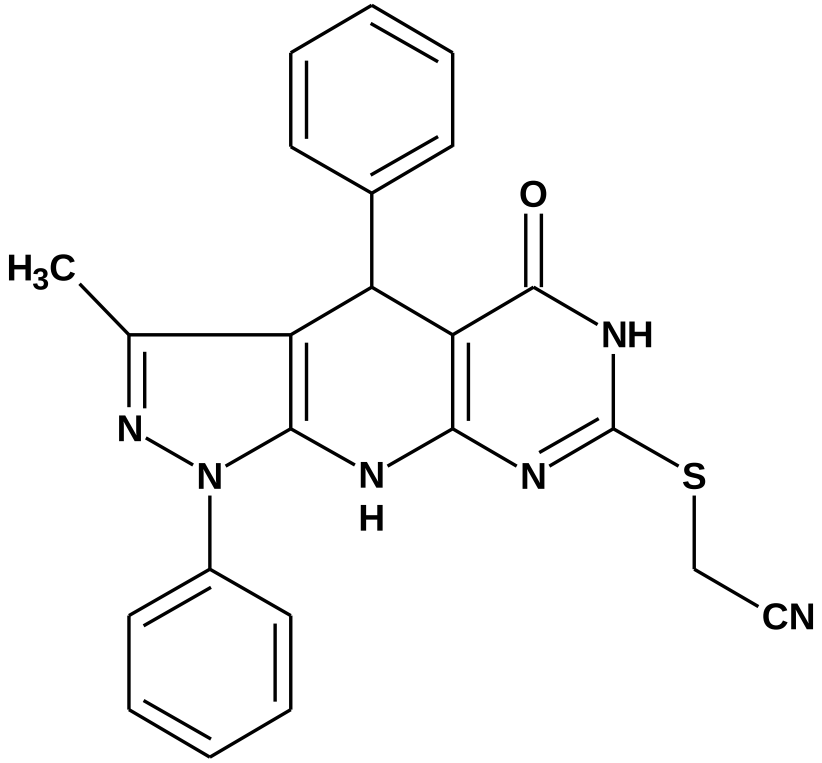

7f

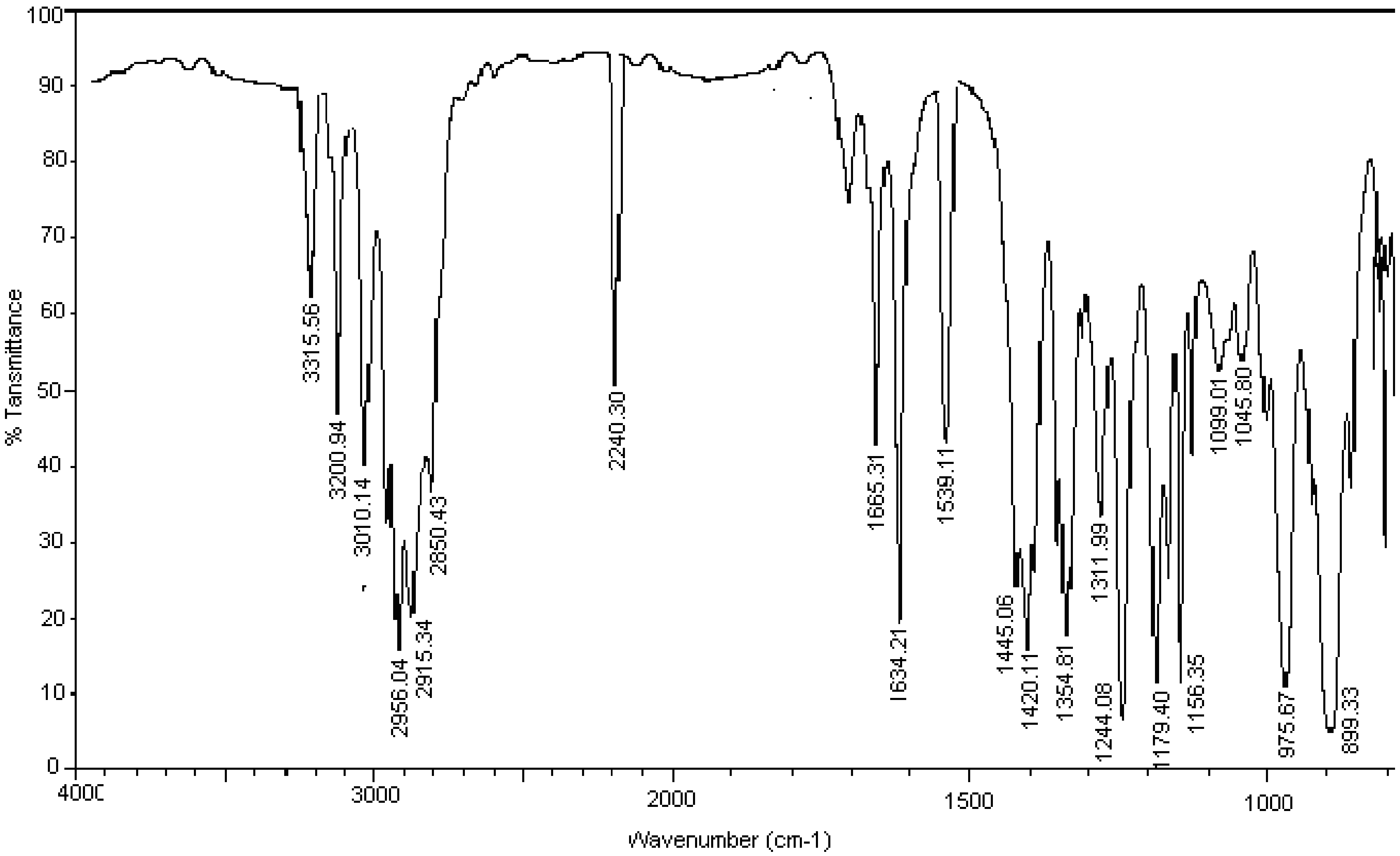

% Tansmittance
Wavenumber (cm-1)
100
90
80
70
60
50
40
30
20
10
0
4000
3000
2000
1500
1000
3315.56
3200.94
3010.14
2956.04
2915.34
2850.43
2240.30
1665.31
1634.21
1539.11
1445.06
1420.11
1354.81
1311.99
1244.08
1179.40
1156.35
1099.01
1045.80
975.67
899.33

^{13}C-NMR spectrum of compound 9

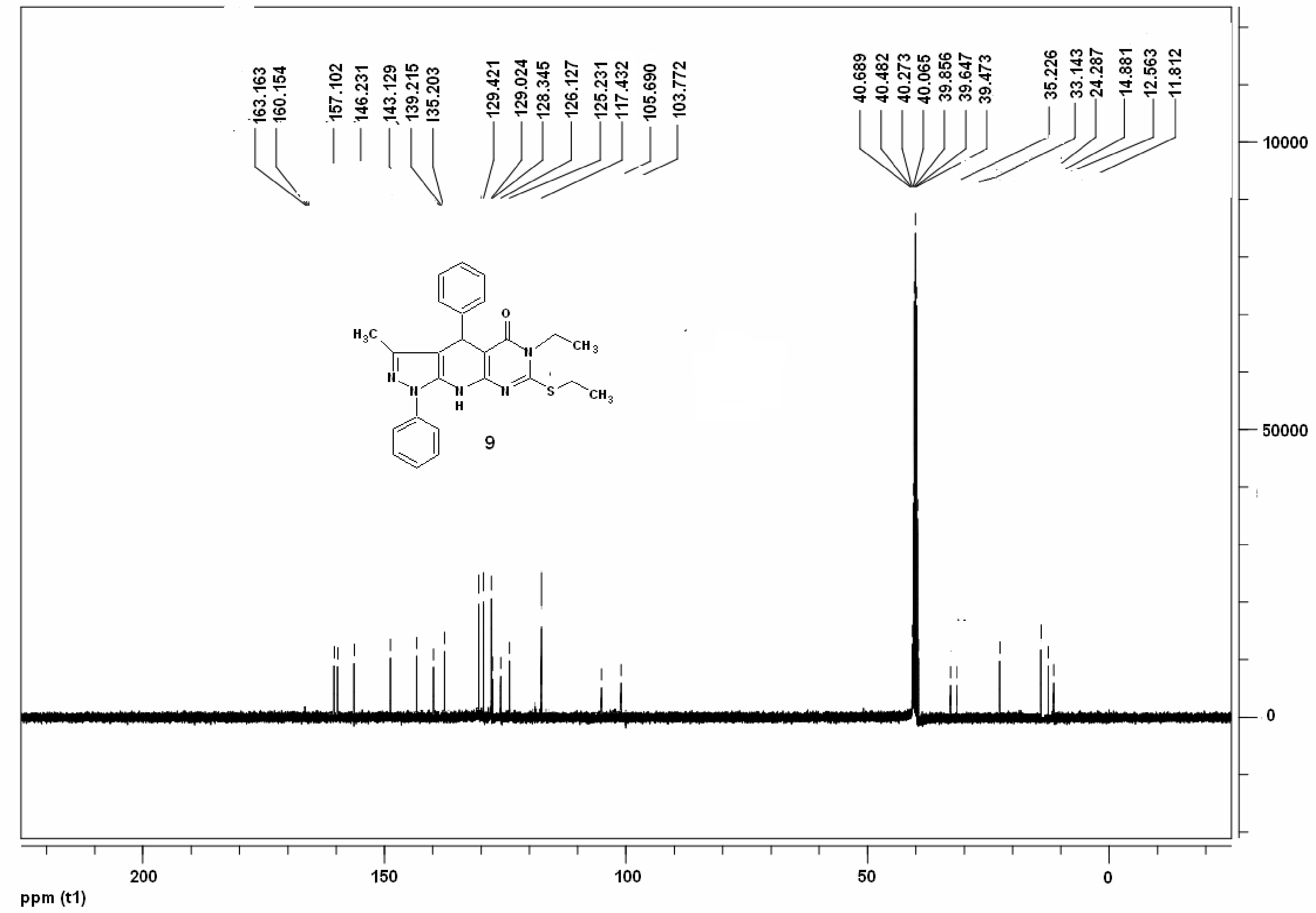

^{1}H-NMR spectrum of compound 11

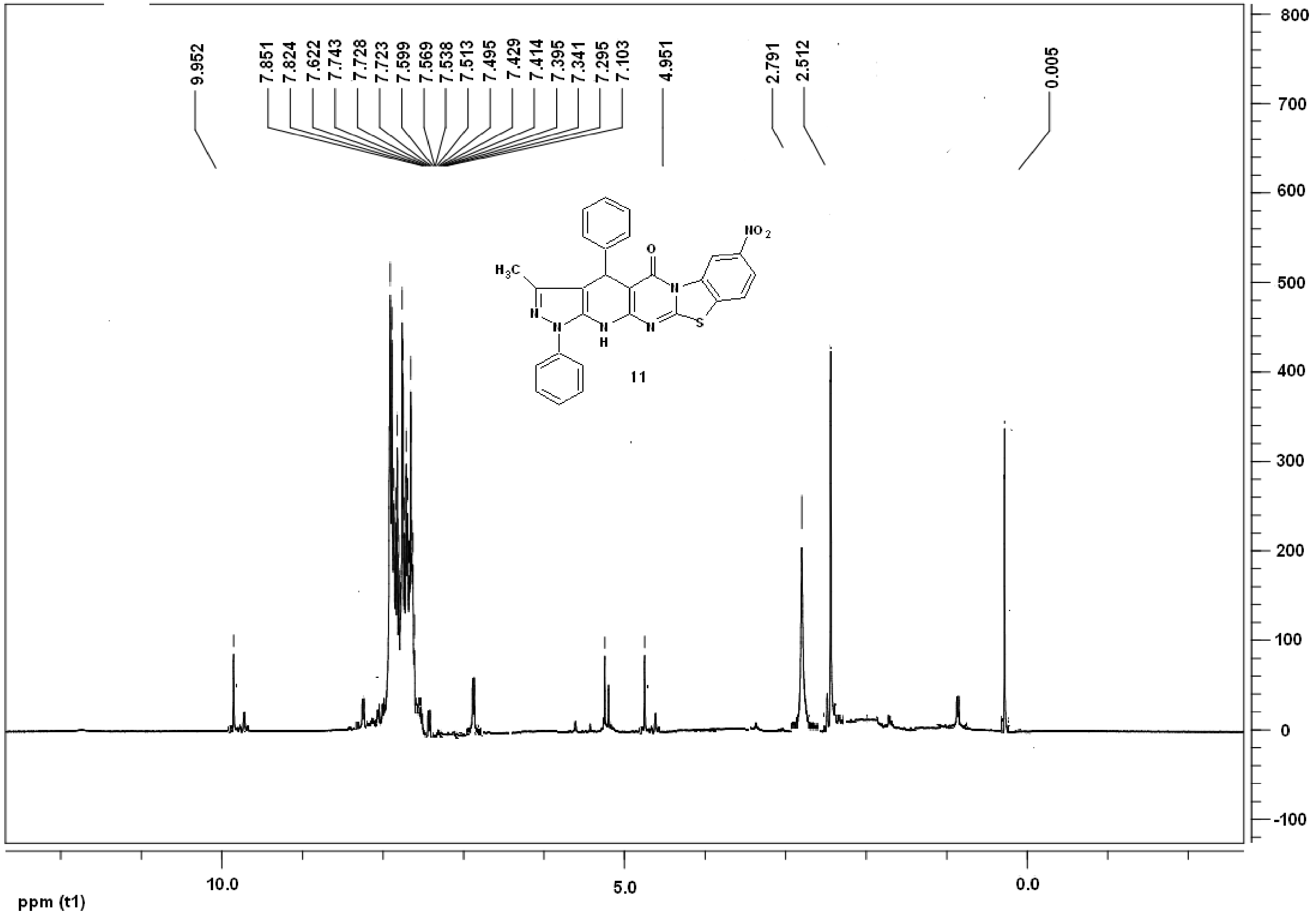

Mass spectrum of compound 11

Assiut University Central lab

8/28/2011 12:55:13 PM Page 1

File: 11ST Date Run : 8/28/2011 Time Run: 12:31:04
Sample: Dr/ Talaat El-Emary
Instrument: JEOL JMS600
Inlet: My Inlet
Ionization mode: EI+
Run By: Souzan
Printed by: Souzan

Scan: 144 **R.T.: 6:00.885**

Base: 506; 38.1%FS TIC: 2533

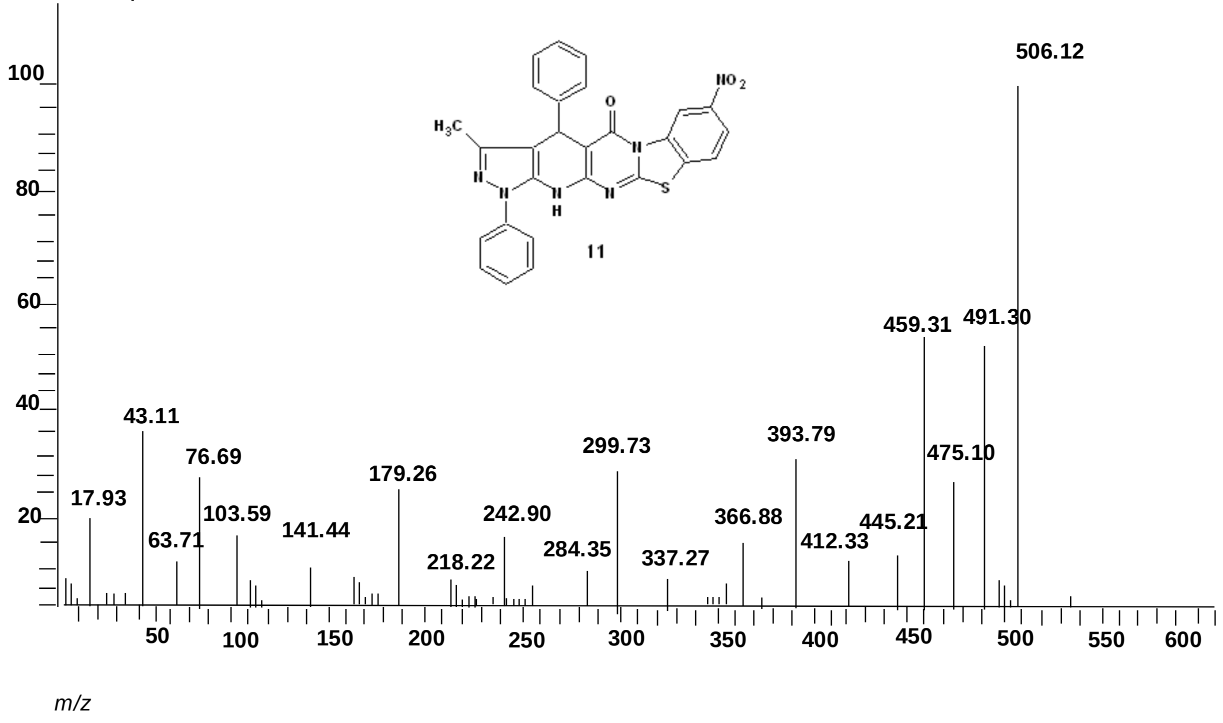

^{13}C-NMR spectrum of compound 20

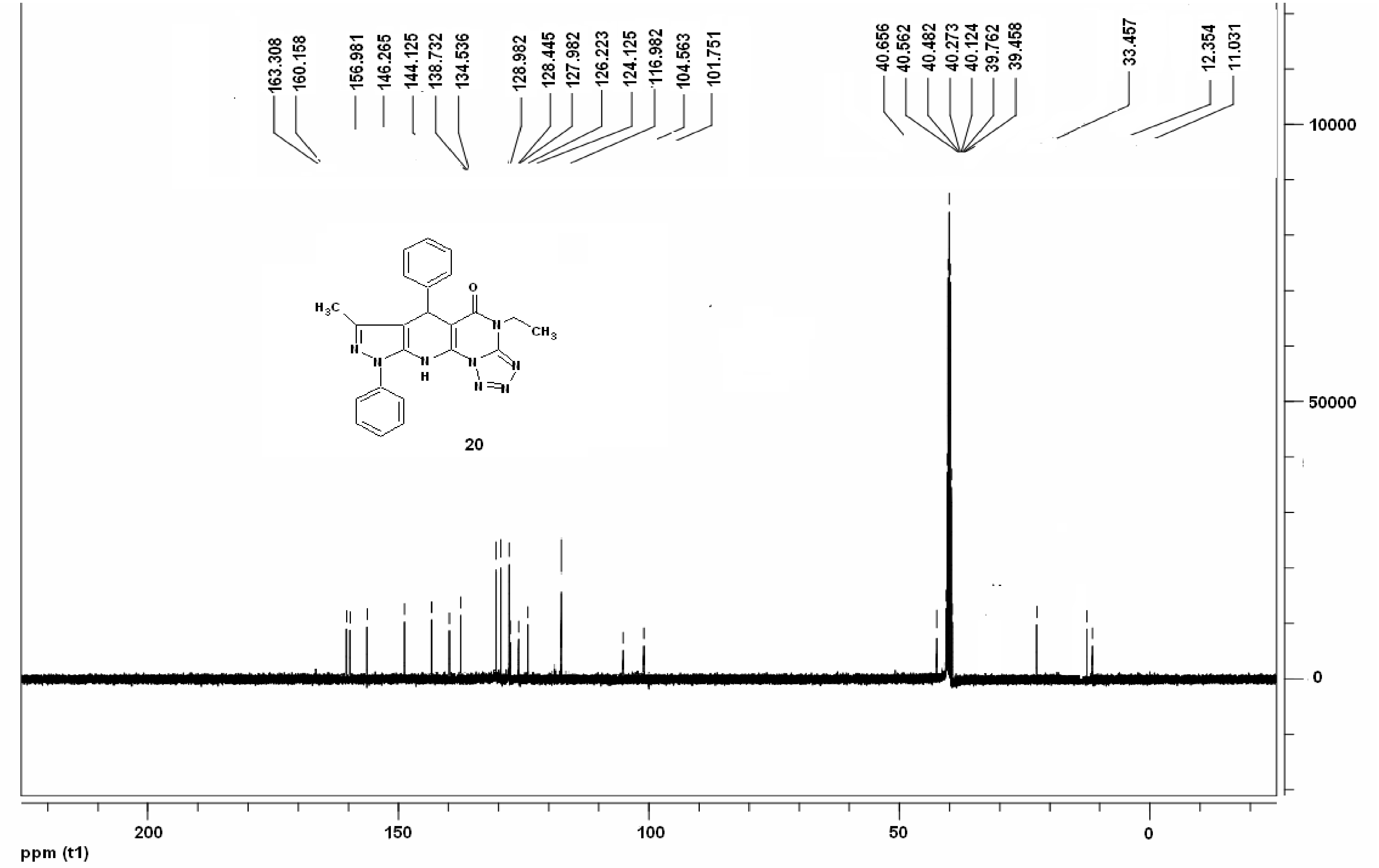

Supplement: Supplementary file 1 [file molecules-17-14464-s001.pdf]
